# An intercultural perspective toward supporting antipsychotic medication adherence in clinical practice

**DOI:** 10.1192/bjb.2022.19

**Published:** 2023-02

**Authors:** Tharun Zacharia

**Affiliations:** South London and Maudsley NHS Foundation Trust, UK

**Keywords:** Antipsychotics, transcultural psychiatry, education and training, schizophrenia, psychotic disorders

## Abstract

In the UK, the incidence of schizophrenia appears highest in Black Caribbean and Black African communities (four- to six-fold that of the White British population). The incidence of psychosis in other minority ethnic groups is also raised, but to a lesser magnitude. Although there are numerous environmental confounding factors, the data stresses the importance of optimising treatment in high-risk (minority) groups. Antipsychotic nonadherence is the most common reason for schizophrenia relapse, and is associated with increased rates of relapse, readmission to hospital and suicide. This article examines available literature to discover how culture can affect antipsychotic nonadherence, and considers culture-based solutions that could enhance antipsychotic adherence. Acknowledging the importance of the therapeutic alliance and sociocultural aspects in antipsychotic adherence, I argue that current cultural competence training provided to clinicians is inadequate. Organisational- and system-level approaches are required to reduce oppressive practise and promote culturally competent, person-centred care.

I am a senior psychiatry trainee and educationalist working in South London. All of my postgraduate psychiatry training has occurred within urban environments, where the incidence of psychosis is higher.^1^ Psychotic illnesses, such as schizophrenia, have a UK lifetime prevalence of 14.5 per 1000 people.^2^ They usually begin in late adolescence/early adulthood,^2^ and can have a devastating functional impact on one's life.^3^

In the UK, the incidence of first-episode psychosis (FEP), non-affective psychosis and schizophrenia are raised across most ethnic minority groups relative to the British population. Following adjustments for several environmental variables, increased incidence of FEP is still noted in Black Caribbean, Black African, Pakistani and mixed ethnicities. However, non-British White or Indian ethnicities do not appear to be at elevated risk of FEP.^4^ Further, minority ethnic groups in the UK are more likely to be admitted compulsorily during an FEP,^5^ as well as during all compulsory detentions in the UK. Of minority groups, Black people are most likely to be compulsorily detained, occurring at a rate that is increasing yearly.^6^ This data highlights the need to think differently about how we support our ethnic minority patients.

Of those with schizophrenia treated with antipsychotics, approximately a third achieve full remission and a further third demonstrate partial remission.^7^ Consensus guidelines generally recommend continuation of antipsychotic medication for 1–2 years after a psychotic illness, to minimise relapse; however, approximately 74% of patients discontinue antipsychotic medication within 18 months.^8^ Similar rates of nonadherence are notably observed in people suffering from a wide range of physical illnesses,^9^ suggesting that lessons learnt may be transferrable across disciplines.

Discontinuation or partial adherence of antipsychotics is associated with a worse prognosis, increased rates of relapse, readmission to hospital and suicide.^10^ Poor adherence is a complex process that can result from a range of factors that encompass the illness, medication and organisation of services, plus attributes of the clinician, patient and caregivers.^11^ However, there are simple and effective practices that the clinician should consider. The therapeutic alliance, in the context of the relationship with the prescriber, is known to be an important determinant of patients’ attitudes toward treatment and adherence to medication.^12^ This alliance remains crucial, as antipsychotic medication adherence is associated with a positive attitude toward medication of both patients and their family, family involvement and illness insight.^13^

## An intercultural perspective

In my own practice, I have struggled to ensure parity of care in supporting patients with antipsychotic adherence when their cultural beliefs about mental illness and medication differ from my own. This article reviews the available literature to discover how culture can affect antipsychotic nonadherence, and consider culture-based solutions that could enhance the therapeutic alliance to support antipsychotic adherence.

Culturally appropriate treatment is advised in the National Institute for Health and Care Excellence guidelines for psychosis and schizophrenia in children and young people, which counsels that ‘services are culturally appropriate’ and that mental health services should ‘ensure that culturally appropriate psychological and psychosocial treatment’ is delivered by ‘competent practitioners’ to ‘children and young people from diverse ethnic and cultural backgrounds’.^14^ Psychological or psychosocial treatment would include supporting medication adherence, and a competent practitioner would have a degree of intercultural competence: specifically, a focus on the interaction and dialogue between different cultures and the need to address healthcare needs within intercultural contexts.^15^ Given the importance of the ‘therapeutic alliance’ in supporting antipsychotic adherence,^10^ a culturally competent practitioner should be able to form a collaborative relationship, in which they can shift between their own cultural lenses and those of the client's, as they co-construct a shared narrative to work toward a shared understanding.^16^

A literature search was completed via the King's College London library catalogue, with the search terms ‘antipsychotic’ AND ‘adherence’ OR ‘compliance’ AND ‘culture’. Foreign language results were excluded for practical reasons (although I note the cultural bias that this could cause), and results from before the year 2000 were excluded to ensure contemporaneous research. Results were then subjectively selected based on topic relevance after review, to explore different cultural aspects of antipsychotic adherence. The search revealed a surprising scarcity of research in the area of cultural interactions on antipsychotic adherence, and specifically, minimal exploration of how culture could affect antipsychotic adherence within a Western healthcare model, and solutions to overcome this cultural barrier.

One cultural barrier may be the differing roles of medical doctors around the world. Unlike physical illness, mental illness is not universally thought of as a biological illness requiring a medical practitioner. In a study from Pakistan, the most common reason for antipsychotic nonadherence was that an ‘alternative treatment pathway was chosen with a traditional faith healer’.^17^ Although faith healing is commonly dismissed as pseudoscience or at least lacks the epistemic warrant to be taken seriously,^18^ it is a common cultural practice worldwide. In low- and middle-income countries, supernatural and psychosocial explanatory models of psychosis appear most prevalent. These models have been found to affect help-seeking behaviour, treatment modalities used and duration of untreated psychosis.^19^

Among British South Asians in the UK, one study suggested that most (55.5%) attributed their psychotic illness to supernatural causes, with few (4.4%) citing a biological cause. The majority (77.7%) held dual explanatory models that combined prescribed medication with seeing a traditional faith healer as a treatment method.^20^

Therefore, there is a close interlink between spiritual beliefs and cultural beliefs/behaviours, requiring greater understanding. Given London's multicultural population, I regularly treat foreign-born patients from diverse spiritual backgrounds. The accepted secular approach often struggles to embrace the culturally important spiritual perspective of a patient's care plan, instigating a barrier to the therapeutic alliance that will affect antipsychotic adherence. Preliminary findings suggest that religiosity is positively associated with treatment adherence in individuals diagnosed with schizophrenia, although religious delusions may also reduce collaboration and treatment adherence.^21^

An effective mental health practitioner might consider working synergistically with faith healers, so long as their practices are not harmful. Alternatively, they may consider incorporating a patient's spiritual needs into their role, while balancing clinically appropriate and societally acceptable practice. Engagement with this unique spiritual domain could lead to a co-constructed shared narrative,^16^ and strengthen the therapeutic alliance. Such integration would remain in keeping with Hippocratic tradition. Although Hippocrates viewed epilepsy as a brain illness, in opposition to the accepted view of a ‘Sacred Disease’,^22^ ancient Greek *Asclepeions* housed both Hippocratic practitioners and religious healers.^23^ Even today, the World Health Organization advocates a need for greater collaboration with ‘informal’ mental healthcare providers, including religious leaders, faith healers and traditional healers, alongside those such as police officers and schoolteachers.^24^ Such collaboration may help to close the mental health treatment gap and offset a larger stigma targeted at traditional healing. but this has largely been studied in low-income countries and more research is required in high-income countries.^25^ A 1997 New Zealand state study by Tamasese et al^36^ explored the views of Samoan elders around the origin of mental illness.^26^ Tamasese et al^36^ noted that elders supported a social origin, more so than a purely biological or spiritual origin.^26^ The study found that Samoan elders believed acculturation and socioeconomic stressors were the major causes of mental unwellness. They did not believe that mental illness was biological. They considered that psychological disruption was caused by alienation from Samoan culture, disturbance of familial relationships, failure to meet family financial obligations and straying from traditional values. They believed that mental illness was a result of spirit possession/spiritual punishment by the imposition of curses for breaching sacred protocols, etiquette or relational arrangements. A literature review proposed that to improve medication adherence, ‘interventions need to be developed to incorporate the traditional belief system of the Samoan New Zealanders and extensive educational initiatives are needed to teach the Samoan New Zealanders about mental illness and medication treatment’.^26^ When treating such cultural groups along the traditional Western biological–psychological–social structure, socially focused care may be required alongside psychoeducation on how (biological) antipsychotics could support individual social well-being.

The study highlights further dissonance between the Western understanding of the aetiology of mental illness and that of some other ethnic groups. Acknowledgement and incorporation into the treatment plan of alternative beliefs on the aetiology of mental illness, alongside educational initiatives on the psychosocial benefits of medications, may support adherence to medication.

The above literature review on Samoan New Zealanders went further to suggest adherence-enhancing interventions that included ‘educational, behavioural, emotional or motivational consumer and family focused interventions and those that target the behaviour of doctors, pharmacists, or nurses’ (Roter et al^39^).^26^ Also, that ‘ethnic populations require the provision of safe and culturally appropriate mental health services (Kowanko et al^37^)^26^ that is offered to consumers in their own language’ (Berg^38^).^26^ Traditional Samoan beliefs were noted to play a significant role in adherence problems in Samoan New Zealanders with psychotic disorders.^26^ It could be postulated that this conclusion may be applicable to ethnic populations within the UK, and an increased focus on incorporating traditional beliefs into holistic, psychosocial antipsychotic adherence planning is required.

A 2013 qualitative study from Taiwan identified four major themes to describe ‘what motivates Taiwanese patients to adhere to antipsychotics: (i) the benefits of antipsychotic medication treatment; (ii) firm and ongoing family support; and the Chinese values of (iii) the concept of filial piety and (iv) hope for the future’.^27^

Many of the above motivators are translatable to UK practice. However, to be effective with minority groups in the UK, a cultural approach would be important. For example, within the above-mentioned study, a benefit of antipsychotics was that they could lead to satisfaction with life; conversely, a participant noted that the illness had led to ‘ … pity that we cannot have three generations living under the same roof … ’.^27^ In traditional UK housing, intergenerational living remains atypical and may not equate to satisfaction with life. Therefore, this highlights the need to develop culturally appropriate meanings for motivators.

The study from Taiwan also highlighted a core motivator that was the Chinese cultural concept of filial piety, which is based on Confucianism, Buddhism and Taoism. It refers to the notion that Chinese children should show extreme respect to their parents, which can be demonstrated by caring for parents, bringing family honour through achievements or at least not being a burden to the family. Participants described how filial piety supported their decision to maintain medication adherence because of wanting to return the parent's love/repay parents, or even having support from elder siblings toward antipsychotic adherence.

Cultural beliefs of honour, respect or obedience toward parents and elder siblings further extend, to some degree, to many other ethnic populations, such as those in the Indian subcontinent. A collectivist Indian family structure supports the physical, spiritual and emotional needs of its members. Interventions that focus on the whole family rather than the individual often result in more gratifying and lasting outcomes.^28^

In the UK, clinicians may support antipsychotic adherence in such ethnic groups by engaging the collective family in antipsychotic education and planning, rather than only the individual; or utilising concepts of filial piety, such as engagement with family elders.

A naturalistic follow-up study from Israel looked at medication adherence in 112 patients with early-episode schizophrenia and found that important predictors of antipsychotic adherence included ‘using the patient's model of illness, patient perception of the need to seek treatment, attitudes toward illness, awareness of medication needs, awareness of social consequences of illness and the patient perceptions of trust in the physician-patient alliance’.^29^

This further strengthens the evidence that UK clinicians must adapt their approach to the patient's cultural model of illness, rather than expect the patient to conform to our own. Insight to illness and medication appear closely tied to antipsychotic adherence, and I would consider if insight might be more easily developed by working within the patient's cultural model of illness. Being mindful of the sociocultural consequences of illness, and working closely with family and other social groups to reduce stigma, should be key goals in reducing nonadherence.^11^ Working in tandem with patients in such a way would likely improve the perception of trust in the physician–patient alliance (therapeutic alliance). These core principles could greatly improve the lives of patients with psychosis, by reducing antipsychotic nonadherence.

## Developing cultural competence in the mental health clinician

Given the importance of the therapeutic alliance and sociocultural aspects of antipsychotic adherence, we can consider that when working with diverse ethnic groups, a degree of cultural competence in the clinician would be vital. I refer to cultural competence in this manner as the explicit use of culturally based care and health knowledge in sensitive, creative and meaningful ways, in nursing and other healthcare professions.^30,31^

**Fig. 1:**
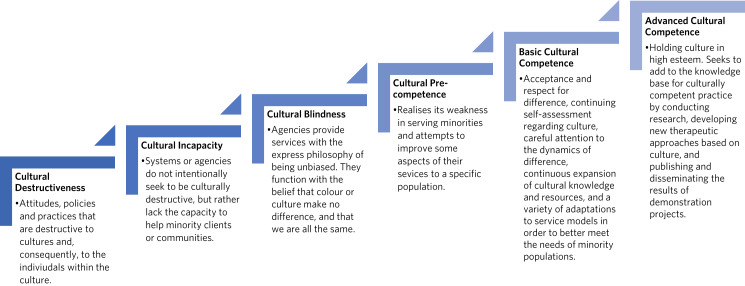
Cultural Competence Continuum (adapted from Cross 1988).^32^

Most National Health Service (NHS) clinicians receive brief and mandatory culture training, and may often sit at stage 3 (cultural blindness) or 4 (cultural pre-competence) within a cultural competence continuum (see Fig. 1).^32^ Predominantly, there is a belief that the Western mental health system is universally applicable, encouraging assimilation but ignoring cultural strengths. E-learning and diversity policies portray services committed to diversity, but often with tokenistic brashness. Increased foreign-born or -trained doctors and medical leaders could play a useful part in helping clinicians toward cultural competence by supporting cross-cultural alliances, effective interaction and personal transformation. However, this is not without its challenges owing to unfair bias compared with their UK counterparts.^33^

A review of NHS mandatory culture competence training seems required, to encourage clinicians toward ‘cultural competence’ or even ‘advanced cultural competence’ (Fig. 1),^32^ where they could deliver a more effective model of person-centred care. Such skills would greatly influence the ability of clinicians to develop holistic therapeutic alliances with patients, reconciling their own and the patient's own cultural attitudes toward mental (and physical) illness and medications, thereby improving antipsychotic adherence.

Cultural diversity initiatives approached from the individual up to the systemic level would best support clinicians toward culture competence. On an individual level, in my own practice, I would argue that a ‘relational/intercultural’ approach would be more beneficial than a ‘cultural literacy’ approach.^34^ Rather than a ‘pre-educated cultural literacy’, a relational approach advocates a position of ‘not knowing’ with a subsequent unique relational learning opportunity. Although time intensive, such a person-centred approach is beneficial in diverse migrant cities such as London. It would be almost impossible to become informed about all cultures that I come into contact with during my work, and I would argue that even with time, one could not provide person-centred care by learning about cultures generically. I question further if it is even possible for someone to become competent in another culture without the experience that comes from ‘lived-in’ full engrossment (language, family, food, religion, etc). Positively, a ‘relational/intercultural’ approach reduces the chance of clinician bias affecting the therapeutic alliance, and lends to an honest and interested, person-centred, patient–physician dynamic. However, this approach is time-consuming, and would remain limited in effectiveness even within my 30 min psychiatric out-patient assessments.

We should also consider ‘organisational’ and ‘systemic’ level approaches to cultural competence,^34^ to consider how we can reduce oppressive practices and promote person-centred care. For example, by challenging the use our rigid models of psychotic illness diagnosis and treatment protocols (which have been validated in Western populations) in minority populations. If cultural diversity is challenged at an organisational level, we could become more adaptable to supporting our patients in tune with their cultural views around mental illness, thereby offering care plans that do not lead them toward cultural dissonance and worsen health disparities. When these treatments involve medication, this way of working would be expected to improve medication adherence, such as with antipsychotics. A challenge is that there is often an ‘internal tension in health organisational culture between those committed to culture competence training (CCT) and those with a tokenistic view’.^35^ A predominant lack of institutional commitment to develop and deliver culture competence training continues to hinder organisational approaches.

## Conclusion

I have reviewed the role of culture in antipsychotic nonadherence, as well as potential motivators. I have considered how intercultural education literature could serve to improve a clinician's cultural competence, leading to strengthening the ability to engage the patient, family and community in a holistic therapeutic alliance, thus supporting a patient in remaining adherent to antipsychotics.

It is overly simplistic to assume that an increase in framing antipsychotic treatments in more culturally competent ways alone would solve the problem of poor antipsychotic adherence. We continue to treat psychosis in all minority populations within our cultural ‘pre-competence’ framework of Western mental health practice. A difficult question that must be asked is, could (and indeed, should) the NHS offer treatments for mental illness within a different cultural framework? I believe that we must be less ethnocentric in the mental health field. Psychosis is a disconnect from the patient's true reality, and care may therefore require culturally appropriate framing. This may include spiritual or sociocultural interventions alongside antipsychotics.

A limitation of this review is the lack of data from immigrant populations within the UK, who may have changed cultural systems to suit their new environment. Assessment of cultural motivators in these specific immigrant populations is needed, as well as testing of generic cultural approaches to antipsychotic adherence that could be replicated on a national scale. Further research is also required to clarify the relationship between increased clinician cultural competence and increased antipsychotic adherence in patients.

There are other important aspects of intercultural doctor–patient relationships that have not been touched upon here, such as the use of interpreters. An interpreter's impact on the clinician's therapeutic alliance, ability to engage the patient's family/social/spiritual domains and convey holistic psychoeducation is undeniably important.

On reflection, all prescribers would likely benefit from advances in cultural competence training to support medication adherence. This is especially true in mental health, because of the diversity of views on illness and the sociocultural impact of illness. Further integration of cultural competence training into the undergraduate medical school curriculum would be an important step toward ensuring prescribers are able to maintain a culturally competent patient relationship, with the advantage of supporting patients to remain adherent to (antipsychotic) medication. This will significantly reduce disease morbidity for patients and their carers, and reduce the significant strain on pressured NHS services. Encouraging both students and clinicians to strive for advanced cultural competence will help to promote a more person-centred, evidence-based, high-quality mental health service for all.

## About the author

**Tharun Zacharia** is a Consultant Psychiatrist with South London and Maudsley NHS Foundation Trust, UK. He is also an Honorary Senior Clinical Lecturer within the Division of Academic Psychiatry at the Institute of Psychiatry, Psychology and Neuroscience, King's College London, UK.
